# For Some Genes, Acetylation/Deacetylation Cycling Is the Real Turn-On

**DOI:** 10.1371/journal.pbio.0030431

**Published:** 2005-11-08

The human genome contains some 20,000–25,000 protein-coding genes, but at any given moment, only a small fraction of them is actively transcribed. The DNA that constitutes a gene is wound around multiple nucleosomes, barrel-shaped protein clusters that serve to organize and protect the DNA. When nucleosomes are packed tightly together, the transcription machinery can't easily reach the gene's promoter segment, where it must bind to begin the transcription process; thus, the gene remains silent. Each nucleosome is a bundle of proteins called histones, with ends (tails) that extend from the nucleosome and are accessible for regulatory modification. Modifications can serve two functions. They may regulate how tightly or loosely nucleosomes pack together, or alternatively, they may function as recognition motifs, allowing other regulatory proteins to be recruited to these nucleosomes. In recent years, therefore, histone modifications have come to be appreciated as a major route for controlling gene expression.

One such modification is the addition of a two-carbon acetyl group to histone H3. Histone acetylation has been widely believed to enhance gene expression. But in this issue, Catherine Hazzalin and Louis Mahadevan show that, for at least some genes, dynamic turnover of acetyl groups on the histone, rather than stable acetylation, is the key to turning on the gene.

Acetyl groups are added to histone H3 by acetyltransferase enzymes, and removed by deacetylase enzymes. The authors showed that when they added a deacetylase inhibitor to cultures of mouse cells, the acetylation level of H3 increased, as they expected. But unexpectedly, they found two modes of sensitivity to deacetylase inhibitors. The majority of H3 was largely insensitive to the presence of the inhibitor. In contrast, the minute fraction of H3 already modified by methyl groups at the fourth amino acid in the tail was immediately and very highly sensitive to deacetylase inhibitors, and picked up new acetyl groups rapidly. Methyl modification at position 4 has previously been associated with increased gene activity.

Acetylation was rapid in genes with multiple methyl groups at position 4, such as c-*fos* and c-*jun*. In contrast, the deacetylase inhibitor did not increase acetylation in β-*globin*, which lacks histone H3 methylated at position 4. For those genes in which acetylation increased, not every nucleosome across the entire gene was equally acetylated, and the pattern of increase appeared to be gene specific. Both c-*fos* and c-*jun* had increased histone H3 acetylation at sites adjacent to the gene's promoter and across the start-of-transcription site, but other regions of each gene were affected differentially between the two.

The authors also showed that three different modifications—methylation at position 4, acetylation at position 9, and addition of a phosphate at position 10—can all occur on the same histone H3 molecule. This tight cluster of modifications is likely to induce significant structural changes in this portion of the molecule, setting the stage for further effects associated with increased gene expression. Another recent paper from this group and others describes the function of some of these modifications at these genes, which is to transiently recruit the phosphate-binding adapter protein 14-3-3 to these nucleosomes.

Finally, the authors asked whether the increase in acetylation brought on by deacetylase inhibition led to an increase in gene activity, in keeping with the prevailing model of gene regulation. The transcription of c-*fos* and c-*jun* can be stimulated by the addition of a chemical inducer. But when cells received the deacetylase inhibitor before, or even up to ten minutes after, inducer treatment, both genes were inhibited. This was due to a direct inhibition of the transcription process, and not from effects on cell signaling or other secondary pathways. Thus, it is turnover, or cycling, of acetylation and deacetylation that is needed to increase expression of these genes.

The mechanism by which continuous acetylation/deacetylation cycling promotes gene expression remains unknown, but these findings add to the complex picture of gene regulation that has emerged since the discovery of histone modification. The hypothesis that there might be a “histone code”—a predictable pattern of modifications invariably associated with increased gene activity—appears to be a simplification, and one that does not, at least in some cases, correspond to the actual dynamic system the cell uses to regulate its genes. —*Richard Robinson*

